# Identification of microRNAs with Dysregulated Expression in Status Epilepticus Induced Epileptogenesis

**DOI:** 10.1371/journal.pone.0163855

**Published:** 2016-10-03

**Authors:** Mykaella Andrade de Araújo, Thalita Ewellyn Batista Sales Marques, Shirley Octacílio-Silva, Carmem Lúcia de Arroxelas-Silva, Marília Gabriella Alves Goulart Pereira, José Eduardo Peixoto-Santos, Ludmyla Kandratavicius, João Pereira Leite, Norberto Garcia-Cairasco, Olagide Wagner Castro, Marcelo Duzzioni, Geraldo Aleixo Passos, Maria Luisa Paçó-Larson, Daniel Leite Góes Gitaí

**Affiliations:** 1 Department of Cellular and Molecular Biology, Institute of Biological Sciences and Health, Federal University of Alagoas, Maceio, Alagoas, Brazil; 2 Department of Morphology, Health and Biological Sciences Center, Federal University of Sergipe, Aracajú, Sergipe, Brazil; 3 Department of Biochemistry, Institute of Biological Sciences, Federal University of Alfenas, Alfenas, Minas Gerais, Brazil; 4 Division of Neurology, Department of Neurosciences and Behavioral Sciences, Ribeirão Preto School of Medicine, University of São Paulo, Ribeirão Preto, São Paulo, Brazil; 5 Department of Physiology, Ribeirão Preto Medical School, University of São Paulo, Ribeirão Preto, São Paulo, Brazil; 6 Department of Physiology and Pharmacology, Institute of Biological Sciences and Health, Federal University of Alagoas, Maceio, Alagoas, Brazil; 7 Department of Genetics, Ribeirão Preto Medical School, University of São Paulo, Ribeirão Preto, São Paulo, Brazil; 8 Department of Cellular and Molecular Biology, Ribeirão Preto Medical School, University of São Paulo, Ribeirão Preto, São Paulo, Brazil; National Institutes of Health, UNITED STATES

## Abstract

The involvement of miRNA in mesial temporal lobe epilepsy (MTLE) pathogenesis has increasingly become a focus of epigenetic studies. Despite advances, the number of known miRNAs with a consistent expression response during epileptogenesis is still small. Addressing this situation requires additional miRNA profiling studies coupled to detailed individual expression analyses. Here, we perform a miRNA microarray analysis of the hippocampus of Wistar rats 24 hours after intra-hippocampal pilocarpine-induced Status Epilepticus (H-PILO SE). We identified 73 miRNAs that undergo significant changes, of which 36 were up-regulated and 37 were down-regulated. To validate, we selected 5 of these (10a-5p, 128a-3p, 196b-5p, 352 and 324-3p) for RT-qPCR analysis. Our results confirmed that miR-352 and 196b-5p levels were significantly higher and miR-128a-3p levels were significantly lower in the hippocampus of H-PILO SE rats. We also evaluated whether the 3 miRNAs show a dysregulated hippocampal expression at three time periods (0h, 24h and chronic phase) after systemic pilocarpine-induced status epilepticus (S-PILO SE). We demonstrate that miR-128a-3p transcripts are significantly reduced at all time points compared to the naïve group. Moreover, miR-196b-5p was significantly higher only at 24h post-SE, while miR-352 transcripts were significantly up-regulated after 24h and in chronic phase (epileptic) rats. Finally, when we compared hippocampi of epileptic and non-epileptic humans, we observed that transcript levels of miRNAs show similar trends to the animal models. In summary, we successfully identified two novel dysregulated miRNAs (196b-5p and 352) and confirmed miR-128a-3p downregulation in SE-induced epileptogenesis. Further functional assays are required to understand the role of these miRNAs in MTLE pathogenesis.

## Introduction

Mesial temporal lobe epilepsy (MTLE) is a common and often medically intractable chronic disease, characterized by spontaneous and recurrent seizures (SRS) [1]. It may develop as a result of a strong cerebral insult, such as status epilepticus (SE) an prolonged epileptic seizure of greater than five minutes or more than one seizure within a five-minute period without the person returning to normal between seizures [2]. SE can cause permanent structural and physiology alterations in the brain, leading to the establishment of an epileptogenic state [3, 4]. These changes include neurodegeneration, neurogenesis, gliosis, axonal damage or sprouting, dendritic plasticity, and inflammation in hippocampus and other limbic structures [5–10]. Although our understanding of epileptogenesis is incomplete, previous research indicates that it is associated with a network-wide reorganization of gene expression in the affected brain [11–20]. Therefore, uncovering the specific factors that lead to the dysregulation of several genes may provide important insights into the epileptogenic process. Recent studies have identified transcription factors driving up- and down-regulation of protein-coding genes after SE [21–24]. Moreover, evidence is emerging that MTLE pathogenesis is controlled by epigenetic factors, including chromatin methylation and small noncoding RNAs [25–29].

MicroRNAs (miRNAs) represent an evolutionarily conserved class of small (22-24 nucleotides) double-strand non-coding RNAs that regulate the expression of target mRNAs by inducing degradation or a reduction in its translational efficiency [30]. Each miRNA can bind to several different transcripts, regulating more than 60% of protein-coding genes [31]. Genes encoding miRNA are transcripted by either RNA polymerase II or III, producing a primary miRNA which is further processed by a Drosha microprocessor complex into stem-loop precursor miRNA (pre-miRNA). After export to the cytoplasm, the pre-miRNA undergoes a final processing by the RNase III enzyme Dicer, generating mature double-stranded miRNA (22-24 nt). One strand is selected and loaded onto the RNA-induced silencing complex (RISC), where Argonaute (Ago) proteins mediate the base-pairing interactions between the miRNA and the 3′ untranslated region (3′ UTR) of target mRNAs resulting in selective post-transcriptional inhibition [32–34].

miRNAs are involved in numerous physiological processes and evidence is emerging that they are dysregulated in acute and chronic diseases of the nervous system such as epilepsy [35–39]. Indeed, since the first report of a change in miRNA expression (miR-132) after a seizure [40], the involvement of miRNAs in epilepsy pathogenesis has become a focus for epigenetic research. Recent work suggests that interfering with miRNA biogenesis increases neuronal excitability and seizure severity [41]. This is consistent with the results of profiling studies, showing that miRNAome undergoes changes in expression during epileptogenesis in both TLE human and animal models [42–57]. However, despite this progress the detailed mechanisms underlying changes in miRNAs and their functional effects during MTLE pathogenesis remain unclear. Part of the reason for this might be the limited number of miRNAs so far identified that show a conserved expression response [58]. Thus, additional miRNA profiling studies are needed to support the cross-comparison of data and to elucidate the role of miRNA in MTLE epileptogenesis. Here, we directly address this shortfall by employing a miRNA microarray approach to screen miRNAs in acute phase of the PILO-induced SE. Furthermore, by using RT-qPCR, we extend expression analysis to other epileptogenic phases, including the chronic phase of epilepsy in human patients.

## Materials and Methods

### Ethics statement

All procedures were performed according to the appropriate ethical guidelines and were approved by the ethical committees of the institutions enrolled. The Brazilian Society for Neuroscience and Behavior approved the experimental model studies, according to international guidelines for ethical use of animals in research, such as those from the Society for Neuroscience. Research Ethics Committee of the Federal University of Alagoas (#011462/2010-83) and Ribeirão Preto Medical School of the University of São Paulo (#195/2005) approved all research protocols performed. All efforts were made to reduce the number of animals used and to avoid unnecessary suffering.

All TLE patients or next-of-kin (for control cases) enrolled in this study read and signed a written Informed Consent Term, previously approved by the Research Ethics Committee. The collection and evaluation of human samples followed the principles of the Declaration of Helsinki, were registered in Brazilian’s Health Ministry and approved by the Research Ethics Committee of the Hospital das Clínicas, where samples were collected (processes HCRP 1781/2010, 15703/2011 and 9370/2003).

### Animal

Experiments were conducted on 48 male adult Wistar rats (200-250 g): 36 were designated for systemic pilocarpine (S-PILO) and respective control groups and were sourced from the main breeding stock of the Federal University of Alagoas; 12 rats were designated for the intra-hippocampal PILO (H-PILO) injection and respective control group and were sourced from the main breeding stock of the University of São Paulo (Ribeirão Preto campus). Rats were kept at 22°C in groups of four per cage with free access to food and water, in a 12-h light/dark cycle (lights on at 08:00 am). Animal health was monitored throughout the experimental period as described previously [59]. No animals presented clinical/behavioral signals of pain or unexpected distress used as humane endpoint criteria for euthanasia.

### S-PILO SE induction

Animals were injected intra-peritoneally (ip) with scopolamine butyl bromide (1 mg/kg) in order to minimize peripheral effects, followed by S-PILO (320 mg/kg; ip) after 30 min. SE was defined as self-sustained seizure behavior or intermittent seizures of less than 5 minutes. PILO administration (110 mg/kg) was repeated after 45 min if the rat did not display seizure behavior. Animals were kept in SE for 90 min before seizure interruption with diazepam (5 mg/kg; ip). All rats presented seizures higher than stage 3 according to the Racine scale [60].

For the chronic group, animals were individually placed in acrylic cages and their behavior was recorded for up to 8 hours per day, during 10 weeks. All the videos were analyzed by two independent observers and the severity of spontaneous seizures was classified according to Racine scale [60]. All of these animals showed two or more spontaneous recurrent seizure with severity scores equal or greater than 3.

In total, three groups of rats were subjected to S-PILO induced SE: i) animals euthanized immediately (0h) after SE reversion (n = 6); ii) animals euthanized 24h after SE reversion (n = 6), and; iii) animals euthanized 11 weeks after SE (Chronic group, n = 6). Naive rats were used as the control group (n = 6).

### H-PILO SE induction

Surgery and microinjections were performed according to Marques et al. [61]. The experimental group (n = 6) was injected with pilocarpine (1.2 mg/μl, 1 μl) and the control group (n = 6) was injected with saline (0.9%; 1 μl). The H-PILO injected animals had 90 minute duration SE, after which seizures were stopped with diazepam (DZP; 5 mg/kg; ip). Control animals were also injected with DZP under the same conditions. These animals were euthanized 24h after SE reversion and only the contra lateral hippocampus was used for gene expression analysis.

### Euthanasia

All the animals were euthanized by decapitation using a guillotine. Hippocampi were immediately removed,dissected on an ice-chilled plate and stored in liquid nitrogen until RNA isolation.

### MTLE patients and controls

All patients were referred for pre-surgical assessment due to drug-resistant epilepsy. Patients were evaluated at the Ribeirão Preto Epilepsy Surgery Program using standardized protocols. Pre-surgical investigation at the Epilepsy Monitoring Unit included detailed clinical history, neurological examination, interictal and ictal video-electroencephalography (Video-EEG), and neuropsychology evaluation. The definition of MTLE followed Engel’s criteria [62]. Clinical data from the MTLE patients were obtained from medical records, and included the following information: presence and age of initial precipitant injury (IPI); estimated monthly frequency of seizures; epilepsy duration; global IQ; verbal and non-verbal memory performance. MTLE specimens were derived from 14 drug-resistant MTLE patients who underwent a standard en bloc anterior temporal resection for seizure control.

Age- and sex-matched tissue from non-epileptic controls (Ctrl, n = 4), obtained in necropsies, were processed and analyzed in the same manner as the surgical cases. All control tissue was collected between 4 ± 1.6 hours after death (maximum of 6 hours postmortem). Underlying diseases causing death were cardiomyopathy, sepsis, or hepatic failure, with no history or evidence of brain pathological abnormalities on postmortem examination of the mesial temporal structures. Surgical and necropsy specimens were cut into 1 cm thick slices, in the coronal plane, immediately frozen and stored at -80°C.

### RNA purification

Total RNA was purified using mirVana total RNA isolation kit (Ambion, Austin, TX, USA) for microarray experiment or Trizol reagent (Invitrogen, CA, USA) for RT-qPCR, following the manufacturers protocol. The quality of total RNA was assessed by analysis of the ratio of 28S to 18S ribosomal RNAs after electrophoresis in 1% agarose gel. For microarray experiments, the miRNA fraction was isolated from 50 μg of total RNA using a flashPAGE Fractionator System (Ambion).

### miRNA microarray analysis

For miRNA labeling, microarray hybridization and data analysis, we followed the procedures described in [63]. The oligo microarray was prepared by spotting the mirVana miRNA Probe Set (Ambion AM 1564V2) on Schott Nexterion E-Scho-1064016 slides (Schott, Mainz, Germany) using a Generation III Array Spotter (Amersham Biosciences-Molecular Dynamics, Sunnyvale, CA, USA). Cy3 labeled miRNAs (for microarray hybridization) were generated using the mirVana miRNA Labeling Kit protocol (Ambion). Labeled miRNA were hybridized with the microarray slides in salt solution for 15 h at 42°C in an Automated Slide Processor ASP (Amersham Biosciences, Sunnyvale, CA, USA). After washing, the slides were scanned using a Generation III array scanner (Amersham Biosciences-Molecular Dynamics) in association with the ArrayVision microarray quantification software (Imaging Research Inc., GE Healthcare, Buckinghamshire, UK). Normalization of data was performed by quantile in the R environment (version 2.11.0) using the AROMA light package (http://www.bioconductor.org). Statistical data were analyzed using the Multiexperiment Viewer (MeV) software (version 3.1 available online at http://www.tm4.org/mev.html). The paired significance analysis of microarrays analysis (SAM available online at http://www.stat.stanford.edu/tibs/SAM), with a false discovery rate (FDR) of 0.05, were used as statistical method to identify the differentially expressed miRNAs [64]. Cluster-TreeView was used to perform the cluster analysis and to construct the miRNA expression profiles [65]. The Microarray data are available in the ArrayExpress database (http://www.ebi.ac.uk/arrayexpress) under accession number E-MEXP-4633.

### RT-qPCR

The RT-qPCR was performed by using TaqMan MicroRNA Assay (Life Tech) to assess the expression of miR-128a-3p (2216 Assay ID); miR-196b-5p (2215 Assay ID); miR-352 (1339 Assay ID), miR-324-3p (579 Assay ID), miR-10a-5p (387 Assay ID). In the reverse transcription (RT) step, cDNA was generated from 1 μg of total RNA using Taqman MicroRNA reverse transcription kit (Life Tech) according to manufacturer’s instructions. Real-time PCR was carried out on a StepOnePlus PCR System (Applied Biosystems) by using TaqMan Universal PCR Master Mix (Life Tech) according to the supplier’s instructions. miR expression was normalized by the combination of U6snRNA and snoRNA for animal analysis as described previously [66], and with U6snRNA and RNU24 for human analysis. Relative fold change was determined by the 2DDCt method [67]. The absence of contamination was confirmed by PCR amplification in the absence of cDNA. Each assay was performed in triplicate and mean values were used for further analysis.

### Statistical Analysis

Statistics were performed using GraphPad Prism 5.00 (GraphPad Software, Inc. San Diego, CA, USA). Unpaired Student’s t-test or Mann Whitney tests were used for comparison of RT-qPCR results related to: i) the microarray validation step (24h versus control), and ii) the human epileptic and non-epileptic analysis. A parametric ANOVA with Bonferroni’s Multiple Comparison Test was used to compare among the different epileptogenesis time points for the expression analysis. For TLE patients, Spearman’s correlation test was used to evaluate the associations between clinical characteristics and microRNA levels, and Student’s t-test were used to evaluate differences in microRNA levels regarding memory scores, surgical outcome (remission vs no-remission), occurrence of initial precipitating injury (IPI), epilepsy focus (right vs left temporal lobe), and sex. Mean differences were considered statistically significant when P<0.05.

### Bioinformatics analysis

Experimentally validated targets of hsa-miR-128a-3p and 196b-5p were compiled from the MicroRNA Target database (miRTarBase)(http://mirtarbase.mbc.nctu.edu.tw/). Pathway analysis was performed by Gene Ontology (GO) to determine the biological significance of these targets, and evaluate their representation (p-value <0.05).

## Results

### microRNAs microarray profile coupled to RT-qPCR validation in hippocampus of rat 24h after H-PILO induced SE

**Fig 1:**
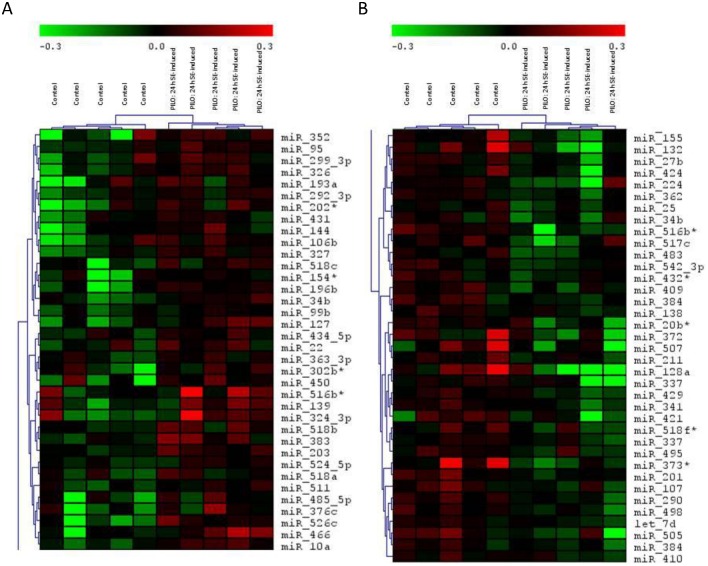
Hierarchical clustering of the 73 miRNA with significantly different expression in 24h post-SE hippocampus versus control experiment. Rows represent individual genes, and columns represent individual samples. The colorgram depicts high (red), average (black) and low (green) expression levels. A) Upregulated microRNAs. B) Downregulated microRNAs. (n = 5 for both H-PILO and control groups).

We used the mirVana miRNA Probe Set covering over 662 miRNAs to identify novel dysregulated miRNAs in rat hippocampi 24h after SE. The miRNA microarray data from the control versus PILO-induced SE experiment showed clear hierarchical clustering (Fig 1). We identified 73 significantly dysregulated microRNAs: 36 (Fig 1A) up-regulated (p < 0.05, FC > 1) and 37 miRNAs (Fig 1B) down-regulated (p < 0.05, FC < 1).

**Fig 2:**
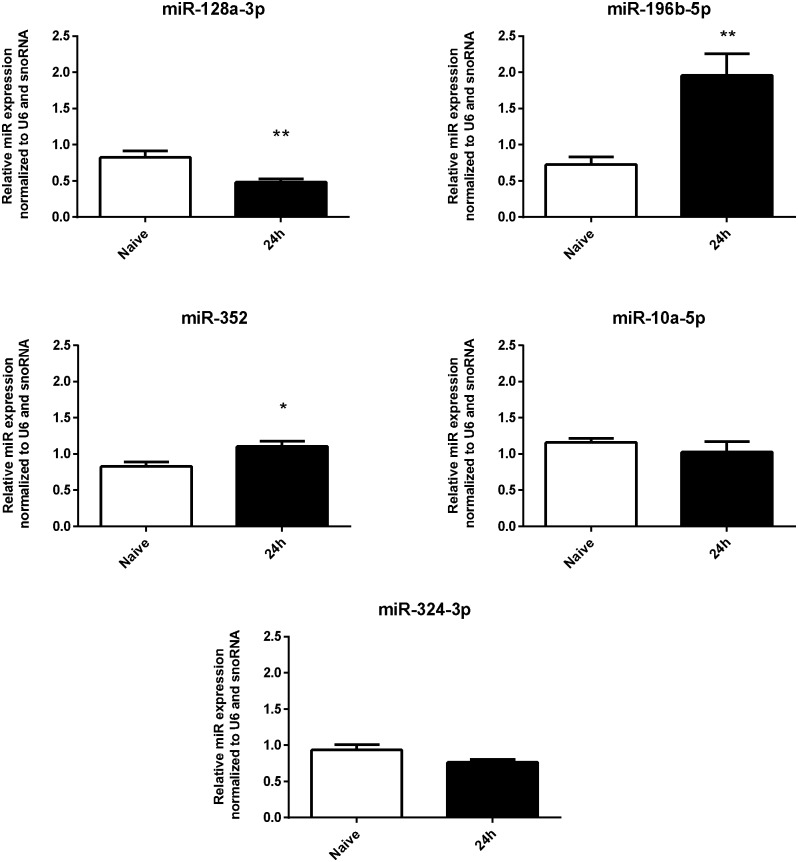
RT-qPCR validation of microarray results. miRs 196b-5p and 352 were significantly increased and miR-128a-3p was significantly reduced in 24h post-SE hippocampus compared with control group. miRs 10a-5p and 324-3p did not show statistically significant differences. (values are mean±SEM, n = 5-6 in each group, *p < 0.05, Unpaired t test).

To verify the accuracy of microarray results we chose a selection of miRNAs from up-regulated (miR-10a-5p, miR-196b-5p, miR-352 and miR-324-3p) and down-regulated (miR-128a-3p) categories for confirmation using the RT-qPCR method. These miRNAs were selected because of their consistent profile among the replicates of each experimental group. We observed that the relative levels of miRs 196b-5p and 352 were significantly higher while the level of miR-128a-3p was significantly reduced in the hippocampi of SE-induced rats in comparison with the control groups (Fig 2). For these miRNAs, the RT-qPCR results were consistent with the microarray analysis.

However, there were no statistical differences between the RT-qPCR for miRs 10a-5p and 324-3p, and the results observed in the microarray analysis could therefore not be validated.

### Expression Patterns of miR-128a-3p, miR-196b-5p and miR-352 in three Stages of S-PILO-SE induced epileptogenic process

**Fig 3:**
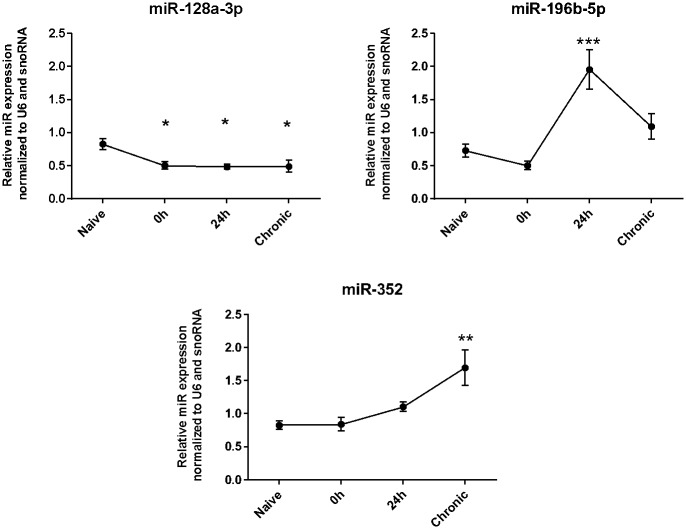
Expression patterns of miR-128a-3p, miR-196b-5p and miR-352 during S-PILO-SE induced epileptogenesis. RT-qPCR measurements of the relative miRs levels (hippocampus) in three different time points after SE-induction (values are mean±SEM, n = 5-6 in each group, *p < 0.05, Bonferroni’s Multiple Comparison Test.

We evaluated whether the selected miRNAs also show a dysregulated hippocampal expression at different time points after S-PILO-induced SE. The analysis was performed by RT-qPCR using tissue samples of control rats and rats that were euthanized immediately (0h), 24h, and 10-12 weeks (chronic phase) after SE (Fig 3). miR-128a-3p transcripts were found to be significantly reduced in post-SE rat hippocampi for all three time points compared with the naïve group. For miR-196b-5p transcript levels, the only significant change was a large increase in the 24h post-SE group compared with all other groups. Finally, miR-352 transcripts were significantly up-regulated in the 24h group compared with naïve, and in the chronic phase group compared with naive or 0h groups.

### Evaluation of miR-128a-3p and miR-196b-5p expression in the hippocampi of epileptic and non-epileptic humans

**Fig 4:**
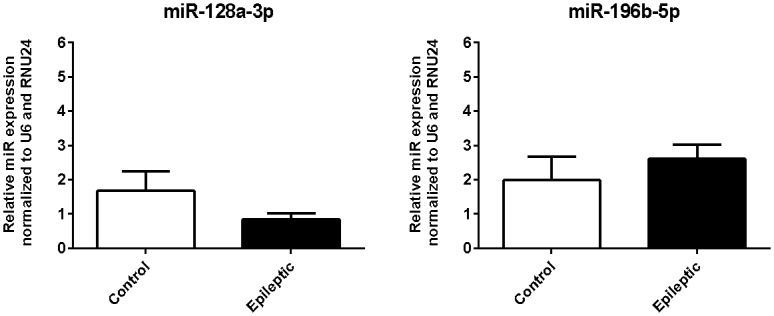
Relative expression of miR-128a-3p and miR-196b-5p in hippocampi from epileptic and non-epileptic humans. RT-qPCR analysis (values are meanSEM, biopsy specimens from TLE-HS patients (n = 10-14) and non-epileptic autopsy samples (n = 4), Mann Whitney test).

Since that only miR-128a-3p and miR-196b-5p are conserved in human and to strengthen the clinical relevance of our study, we assessed whether these miRs expression are dysregulated in human tissue samples. To achieve this, we compared hippocampal specimens from TLE patients with hippocampal sclerosis (HS) against hippocampal samples from non-epileptic individuals. Although the RT-qPCR results were not significantly different for the two miRNAs, transcript levels showed similar downward (miR-128a-3p) or upward (miR-196b-5p) trends to those observed in the animal model (Fig 4).

In TLE patients, there was no correlation between miR-128a-3p or miR-196b-5p levels and age at surgery, seizure frequency, age at first seizure, age at seizure recurrence, or global IQ (S1 Table). Moreover, there was no difference in the expression of miR-128a-3p or miR-196b-5p when comparing surgical outcome, verbal memory score, non-verbal memory score, IPI, epileptic focus, or sex (S2 Table).

### Bioinformatics analysis of miR-128a-3p and 196b-5p targets

To gain insights into functional links between changes in the expression of miR-128a-3p and 196b-5p and epileptogenesis, we performed an analysis of cellular pathways potentially enriched in these miRs targets. We identified 490 and 139 validated targets of hsa-miR-128a-3p and 196b-5p, respectively, deposited in miRTarBase (S3 and S4 Tables). The pathways significantly over-represented in these miRNA targets are listed in Tables 1 and 2, where enrichment test p values for each GO term are indicated.

**Table 1:** GO Pathway significantly overrepresented among the miR-128a-3p validated targets.

Term	Description	P-value
(*P*06959)	CCKR signaling map	3.20E-04
(*P*00027)	Heterotrimeric G-protein signaling pathway-Gq alpha and Go alpha mediated pathway	8.51E-04
(*P*00047)	PDGF signaling pathway	1.04E-03
(*P*00059)	p53 pathway	1.38E-03
(*P*00048)	PI3 kinase pathway	2.22E-03
(*P*06664)	Gonadotropin-releasing hormone receptor pathway	4.67E-03
(*P*00032)	Insulin/IGF pathway-mitogen activated protein kinase kinase/MAP kinase cascade	8.34E-03
(*P*04398)	p53 pathway feedback loops 2	9.20E-03
(*P*00020)	FAS signaling pathway	9.23E-03
(*P*00005)	Angiogenesis	1.03E-02
(*P*05911)	Angiotensin II-stimulated signaling through G proteins and beta-arrestin	1.46E-02
(*P*00033)	Insulin/IGF pathway-protein kinase B signaling cascade	1.72E-02
(*P*00034)	Integrin signalling pathway	1.78E-02
(*P*00029)	Huntington disease	1.92E-02
(*P*04385)	Histamine H1 receptor mediated signaling pathway	2.16E-02
(*P*00056)	VEGF signaling pathway	2.98E-02
(*P*00049)	Parkinson disease	3.35E-02
(*P*04380)	Cortocotropin releasing factor receptor signaling pathway	4.15E-02
(*P*00018)	EGF receptor signaling pathway	5.00E-02

**Table 2:** GO Pathway significantly overrepresented among the miR-196b-5p validated targets.

Term	Description	P-value
(*P*00010)	B cell activation	1.70E-04
(*P*00028)	Heterotrimeric G-protein signaling pathway-rod outer segment phototransduction	1.99E-04
(*P*06664)	Gonadotropin-releasing hormone receptor pathway	1.44E-03
(*P*06959)	CCKR signaling map	1.48E-03
(*P*00006)	Apoptosis signaling pathway	1.80E-03
(*P*00053)	T cell activation	4.88E-03
(*P*00026)	Heterotrimeric G-protein signaling pathway-Gi alpha and Gs alpha mediated pathway	6.25E-03
(*P*02773)	S-adenosylmethionine biosynthesis	2.08E-02
(*P*00047)	PDGF signaling pathway	2.14E-02
(*P*00019)	Endothelin signaling pathway	2.31E-02
(*P*05911)	Angiotensin II-stimulated signaling through G proteins and beta-arrestin	3.11E-02
(*P*00036)	Interleukin signaling pathway	3.22E-02
(*P*04378)	Beta2 adrenergic receptor signaling pathway	4.19E-02
(*P*04377)	Beta1 adrenergic receptor signaling pathway	4.19E-02
(*P*04373)	5HT1 type receptor mediated signaling pathway	4.19E-02
(*P*00034)	Integrin signalling pathway	4.70E-02

## Discussion

Emerging data show that status epilepticus (SE) triggers a reorganization of miRNA expression in the brain [58, 68, 69]. We employed microarray analyses to identify dysregulated miRNAs in SE-induced epileptogenesis, detecting 73 miRNAs (37 downregulated and 36 upregulated) with differential expression in 24h post-SE rat hippocampi (Fig 1).To strengthen the discussion of our own data in the context of previously published studies, we performed a cross-comparison using data from large-scale profile of miRNA responses during the epileptogenic process, which are available in the EpimiR database [70]. Only 15 of the miRNAs identified in our analysis (miRs 518c-3p, 516b-3p, 518b, 524-5p, 518a-3p, 362-5p, 517c-3p, 409-3p, 20b-3p, 372-3p, 507, 421-5p, 518f-5p, 373-5p, 201-5p) had not yet been linked to epilepsy/seizure. In order to identify which of our miRNAs had a conserved response in acute phase of SE-induced epileptogenesis, we further restricted the comparison to studies with profiled miRNA responses in the 24 h after SE induction [47, 49, 54, 57]. A total of 24 miRNAs detected in our study have also been identified in previous studies. Moreover, 14 of our miRNAs showed a change in expression that was in the same direction in at least one of these profiling studies. Interestingly, despite the acknowledged difficulties of cross-comparing data derived from different experimental protocols, most of the miRNAs (22-3p, 139-5p, 144-3p, 203-3p, 326-3p, 431-5p, 196b-5p, 25-3p, 337-3p, 495-3p, 34b-5p, 542-3p) showed overlap with other post-SE models such as lithium-PILO, kainite or electrical stimulation. These common miRNAs are particularly attractive for further functional investigation because they likely to be disease, rather than model-specific. On the other hand, the response of certain miRNAs (e.g. miR-146 and miR-134) following SE contrasts with results from other studies, probably due to specificities of the H-PILO or technical limitations inherent in the use of a large-scale experimental approach. Indeed, there are few overlaps among miRNAs profiles generated by using high-throughput platforms, even considering those from the same model. One reason for this is that miRNA microarray data need to undergo an individual validation step. We observed that, from the subset of five miRNAs (10a-5p, 128a-3p, 196b-5p, 324-3p and 352) selected for individual RT-qPCR analysis, three (miRs-128a-3p, 196b-5p and 352) presented consistent results with the microarray analysis (Fig 1). These were consequently chosen for more extensive expression analysis during the epileptogenic process.

We used RT-qPCR to measure changes in expression of miR-128a-3p, 196b-5p and 352 expression at three time periods (0h, 24h and 10-12 weeks) after S-PILO induced SE. We observed: i) down-regulation of miR-128a-3p in the three stages of epileptogenesis; ii) up-regulation of miR-196b-5p only at 24h post-SE, and; iii) up-regulation of miR-352 in both 24h and chronic post-SE time points. It is important to highlight that the results obtained with S-Pilo model were similar to those observed for the H-Pilo model. Thus, the potential differences between the activated/suppressed pathways in the induction of seizures in these two models do not seem to have a differential impact on the expression of these miRs.

Despite of miR-128a-3p expression is dysregulated in hippocampus of epileptic rats (chronic group), we did not observe a statistical significant differences of its levels in hippocampal biopsy specimens from TLE-HS patients comparing with non-epileptic autopsy samples. This finding could be a consequence of the fundamental physiological differences between rats and humans. Alternatively, it could be due to various confounding factors that are common in the analysis of differential expression using human specimens, such as ethnicity, BMI, lifestyle, and other individual characteristics. Tissue origin (i.e. biopsy or autopsy) is also a critical issue because autopsy delay would tend to confound the difference between control and TLE hippocampal biopsy samples [42, 46]. Indeed, this could explain why our RT-qPCR results show that miR transcript levels tend to change in a similar direction to that observed in the animal model. However, whether dysregulation of miRs 128a-3p and 196b-5p can be extended to epileptic patients requires further clarification.

In favor of that is our finding of hsa-miRs 128a-3p and 196b-5p target genes enrichment in common signaling pathways that have been associated to the molecular mechanisms underlying seizures and epilepsy. For instance, we observed that the integrin signalling pathway that was found significantly over-represented for both miR-128a-3p and 196b-5p targets (Table 3) plays a role in several neuropathological processes of epileptogenesis [71–74].

**Table 3:** GO Pathway commonly overrepresented among the miR-128a-3p and 196b-5p validated targets.

GO	Description
(*P*06959)	CCKR signaling map
(*P*00027)	Heterotrimeric G-protein signaling pathway-Gq alpha and Go alpha mediated pathway
(*P*00047)	PDGF signaling pathway
(*P*06664)	Gonadotropin-releasing hormone receptor pathway
(*P*05911)	Angiotensin II-stimulated signaling through G proteins and beta-arrestin
(*P*00034)	Integrin signalling pathway

It is also known that miR-128 is highly enriched in adult mouse and human brains, and has recently been linked to epilepsy [75]. Similar to our observation, prominent down-regulation of miR-128 has been recorded in the acute and chronic phase of Litio-PILO induced epileptogenesis [75]. Moreover, it has been shown that the absence of miR-128 expression in miR-128-2−/− mice causes seizure-induced death, which is prevented by its overexpression [76]. Although the mechanism of action remains a source of speculation, these data strongly suggest an anti-epileptogenic role for miR128a. In fact, the bioinformatics analysis of the validated targets of hsa-miR-128 showed a significantly over-representation of pathways such as P53, Insulin/IGF pathway-mitogen activated protein kinase kinase/MAP kinase cascade (Table 1), which are enhanced after SE insult.

In contrast, the involvement of miR-196b-5p in epileptogenesis and epilepsy is unknown. miR-196b has been identified as a regulator of tumorigenesis and its overexpression may be associated with the occurrence of preoperative seizures in low-grade gliomas [77]. Significantly over-represented signaling pathways for miR-196b-5p include apoptosis signaling (Table 2). A negative regulation of anti-apoptotic genes, by the up-regulation of miR-196b, could contribute to the cell death seen after the SE. In fact, the MiRTar tool indicated BCL2 as a potential target of miR-196b-5p by imunohistochemical-based validation. The anti-apoptotic BCL2 gene is down-regulated in the hippocampus of rats 48 hours after KA-induced SE [78], which may contribute to neurodegeneration occurring during epileptogenesis. However, the potential involvement up-regulation of miR-196b-5p in MTLE pathogenesis by regulating the BCL2 will require further study. On the other hand, a recent study showed that the expression of PI3K/AKT/mTOR proteins and mRNAs were increased following upregulation of the expression of miR-196b-5p in cancer cells [79]. Interesting some member of this pathway (P-Akt and p70S6K) are also increased in the hippocampi of children with MTLE and the negative modulation of the PI3K/Akt/mTOR signaling pathway has been suggested as a novel therapeutic target for the treatment of MTLE [80]. Further study may show whether the up-regulation of miR-196b-5p contribute to activation of PI3K/Akt/mTOR signaling pathway in MTLE pathogenesis.

Identifying a potential link between miR-352 and epilepsy is challenging because the biological role of this miR is unknown. An additional complication is the absence of an experimentally validated target for miR-352. The only functional assay indicated that this miRNA targets the HEXB gene and may regulate lysosomal-associated proteins following ischemic stroke [81]. Hexb is the beta subunit of the lysosomal enzyme beta-hexosaminidase. When mutated, this gene can cause Sandhoff disease—a progressive neurodegenerative disorder characterized by accumulation of GM2 gangliosides that can correlate with seizures [82]. This suggests that an investigation of HEXB and the identification of other direct targets would be a productive next step towards uncovering the role of miR-352 in epileptogenesis. A recent study showed that SE results in significant accumulation of autophagosome- and lysosome-associated proteins in neurites [83]. The authors suggested that lysosomal/autophagic mechanisms reflect an attempt to survive the epileptic insult by breaking down non-essential components. Further functional studies are required to investigate the miR-352 involvement in autophagy dynamics during epileptogenesis.

## Supporting Information

S1 Table Correlation between clinical characteristics and miR-128a-3p and miR-196b-5p expression levels in TLE patients. (DOCX)

S2 Table Levels of miR-128a-3p and miR-196b-5p in accordance to memory performance, epilepsy remission, IPI, epileptic focus or sex. (DOCX)

S3 Table Validated targets of miR-128a-3p based on miRTarBase. (XLSX)

S4 Table Validated targets of miR-196b-5p based on miRTarBase. (XLSX)
