# Supplementary material for: Identification of microRNAs with Dysregulated Expression in Status Epilepticus Induced Epileptogenesis
Source: PLoS One. 2016 Oct 3;11(10):e0163855. doi: 10.1371/journal.pone.0163855 (PMC5047645; doi:10.1371/journal.pone.0163855)
Supplement: S1 Table — (DOCX) [file pone.0163855.s001.docx]

**S1 Table.** **Correlation between clinical characteristics and miR-128a-3p and miR-196b-5p expression levels in TLE patients.**

|  | miR-128a-3p | | miR-196b-5p | |
| --- | --- | --- | --- | --- |
|  | Correlation coefficient** | p-value | Correlation coefficient | p-value |
| Age at surgery* | 0.171 | 0.607 | -0.0502 | 0.860 |
| Seizures per month | 0.150 | 0.656 | 0.146 | 0.635 |
| Age at first seizure* | -0.177 | 0.607 | -0.443 | 0.143 |
| Seizure recurrence* | 0.323 | 0.346 | -0.302 | 0.329 |
| Global IQ | -0.224 | 0.512 | -0.414 | 0.173 |

* Age in years

** Spearman’s test

** SEM = standard error of mean
