# Supplementary material for: Identification of microRNAs with Dysregulated Expression in Status Epilepticus Induced Epileptogenesis
Source: PLoS One. 2016 Oct 3;11(10):e0163855. doi: 10.1371/journal.pone.0163855 (PMC5047645; doi:10.1371/journal.pone.0163855)
Supplement: S2 Table — (DOCX) [file pone.0163855.s002.docx]

**S2 Table.** **Levels of miR-128a-3p and miR-196b-5p in accordance to memory performance, epilepsy remission, IPI, epileptic focus or sex.**

|  |  | miR-128a-3p |  | miR-196b-5p | |
| --- | --- | --- | --- | --- | --- |
|  |  | Mean ± SEM | p-value | Mean ± SEM** | p-value |
| Verbal memory | Average or above | 0.929 ± 0.381 | 0.733 | 2.580 ± 0.887 | 0.940 |
|  | Below average | 0.800 ± 0.164 |  | 2.647 ± 0.436 |  |
| Non-verbal memory | Average or above | 0.609 ± 0.192 | 0.381 | 1.580 ± 0.232 | 0.127 |
|  | Below average | 0.956 ± 0.225 |  | 2.973 ± 0.463 |  |
| Epilepsy remission | Yes | 0.916 ± 0.277 | 0.668 | 3.195 ± 0.605 | 0.152 |
|  | No | 0.755 ± 0.138 |  | 2.054 ± 0.417 |  |
| IPI* | Yes | 0.702 ± 0.194 | 0.411 | 2.451 ± 0.474 | 0.725 |
|  | No | 1.001 ± 0.284 |  | 2.749 ± 0.605 |  |
| Epileptic focus | Right | 0.949 ± 0.296 | 0.598 | 2.451 ± 0.620 | 0.678 |
|  | Left | 0.755 ± 0.193 |  | 2.798 ± 0.523 |  |
| Sex | Female | 0.366 ± 0.067 | 0.054 | 2.046 ± 0.435 | 0.226 |
|  | Male | 1.060 ± 0.192 |  | 3.038 ± 0.566 |  |

*initial precipitating injury

** SEM = standard error of mean
